# Comparison of exosomes secreted by induced pluripotent stem cell-derived mesenchymal stem cells and synovial membrane-derived mesenchymal stem cells for the treatment of osteoarthritis

**DOI:** 10.1186/s13287-017-0510-9

**Published:** 2017-03-09

**Authors:** Yu Zhu, Yuchen Wang, Bizeng Zhao, Xin Niu, Bin Hu, Qing Li, Juntao Zhang, Jian Ding, Yunfeng Chen, Yang Wang

**Affiliations:** 1 Department of Orthopedic Surgery, Shanghai Jiao Tong University Affiliated Sixth People’s Hospital, 600 Yishan Road, Shanghai, 200233 China; 2 Institute of Microsurgery on Extremities, Shanghai Jiao Tong University Affiliated Sixth People’s Hospital, 600 Yishan Road, Shanghai, 200233 China

**Keywords:** Exosomes, Induced pluripotent stem cell-derived mesenchymal stem cell, Synovial membrane-derived mesenchymal stem cell, Osteoarthritis

## Abstract

**Background:**

Osteoarthritis (OA) is the most common joint disease worldwide. In the past decade, mesenchymal stem cells (MSCs) have been used widely for the treatment of OA. A potential mechanism of MSC-based therapies has been attributed to the paracrine secretion of trophic factors, in which exosomes may play a major role. In this study, we aimed to compare the effectiveness of exosomes secreted by synovial membrane MSCs (SMMSC-Exos) and exosomes secreted by induced pluripotent stem cell-derived MSCs (iMSC-Exos) on the treatment of OA.

**Methods:**

Induced pluripotent stem cell-derived MSCs and synovial membrane MSCs were characterized by flow cytometry. iMSC-Exos and SMMSC-Exos were isolated using an ultrafiltration method. Tunable resistive pulse-sensing analysis, transmission electron microscopy, and western blots were used to identify exosomes. iMSC-Exos and SMMSC-Exos were injected intra-articularly in a mouse model of collagenase-induced OA and the efficacy of exosome injections was assessed by macroscopic, histological, and immunohistochemistry analysis. We also evaluated the effects of iMSC-Exos and SMMSC-Exos on proliferation and migration of human chondrocytes by cell-counting and scratch assays, respectively.

**Results:**

The majority of iMSC-Exos and SMMSC-Exos were approximately 50–150 nm in diameter and expressed CD9, CD63, and TSG101. The injection of iMSC-Exos and SMMSC-Exos both attenuated OA in the mouse OA model, but iMSC-Exos had a superior therapeutic effect compared with SMMSC-Exos. Similarly, chondrocyte migration and proliferation were stimulated by both iMSC-Exos and SMMSC-Exos, with iMSC-Exos exerting a stronger effect.

**Conclusions:**

The present study demonstrated that iMSC-Exos have a greater therapeutic effect on OA than SMMSC-Exos. Because autologous iMSCs are theoretically inexhaustible, iMSC-Exos may represent a novel therapeutic approach for the treatment of OA.

**Electronic supplementary material:**

The online version of this article (doi:10.1186/s13287-017-0510-9) contains supplementary material, which is available to authorized users.

## Background

Osteoarthritis (OA) is the most common joint disease worldwide, affecting an estimated 10% of men and 18% of women over 60 years of age, making it a major healthcare burden on society [1]. Because of the lack of blood supply to articular cartilage and because chondrocytes are highly differentiated cells with poor proliferative and migration potential [2, 3], the treatment of OA has always been problematic. Advances in stem cell transplantation therapy have shown promise in treating OA. In the past decade, mesenchymal stem cells (MSCs) such as bone marrow-derived MSCs (BMSCs) [4–6] and adipose-derived MSCs (AMSCs) [7, 8] have been used widely for the treatment of OA. However, many disadvantages of stem cell transplantation therapy still remain to be overcome, including the risk of tumor formation, ethical concerns, and graft rejection, among others [9]. In addition, there are challenges associated with the proper handling of stem cells and the optimal storage conditions for maintaining cell viability and vitality. Consequently, there is a need to develop new strategies to overcome the disadvantages of cell transplantation therapy.

The efficacy of many MSC-based therapies has been attributed to the paracrine secretion of trophic factors, and exosomes may play a major role in mediating tissue repair [10, 11]. Exosomes derived from different stem cells have been demonstrated to facilitate tissue repair in the skin [12], limbs [13], heart [14], and other tissues. To our knowledge, however, the effect of MSC exosomes on OA repair has not been investigated.

An important issue in developing MSC exosome therapy for OA is determining the ideal cell type for exosome isolation. Recently, researchers have demonstrated that synovial membrane-derived MSCs (SMMSCs) can inhibit OA progression [15, 16]. SMMSCs are particularly well suited for cartilage repair because the synovium and cartilage originate from a common pool of cells during the development of synovial joints [17, 18], suggesting that SMMSCs are developmentally more closely related to chondrocytes than to other MSCs. Moreover, SMMSCs have been reported to more readily undergo chondrogenesis than BMSCs and AMSCs [19]. However, SMMSCs are hard to obtain, and synovial membranes can only be obtained through an invasive approach.

As an alternative source of stem cells, human induced pluripotent stem cells (iPSCs) can be induced from patient-specific adult somatic cells, and are similar to embryonic stem cells (ESCs) in terms of morphology, self-renewal, and differentiation capacity [20, 21]. Because they are patient specific, iPSC-derived MSCs (iMSCs) can theoretically eliminate the need for immunosuppression in the recipient. Autologous iMSCs could therefore be considered an inexhaustible source of MSCs that could be used to meet as yet unmet clinical needs. Moreover, when compared with adult MSCs, human iMSCs have been demonstrated to be superior with regard to cell proliferation, immunomodulation, cytokine profile, generation of exosomes capable of modulating the microenvironment, and bioactive paracrine factor secretion [22]. To our knowledge, however, whether exosomes secreted by synovial membrane MSCs (SMMSC-Exos) or exosomes secreted by induced pluripotent stem cell-derived MSCs (iMSC-Exos) are better for the treatment of OA has not yet been reported.

In this study, we aimed to compare the effectiveness of exosomes isolated from either SMMSCs (SMMSC-Exos) or iMSCs (iMSC-Exos) on the treatment of OA. We found that iMSC-Exos had a superior therapeutic effect compared with SMMSC-Exos in a mouse model of collagenase-induced OA. Further in-vitro studies demonstrated that iMSC-Exos were more effective in stimulating chondrocyte migration and proliferation than SMMSC-Exos. Our results suggest the possible therapeutic use of exosomes as a novel treatment for OA.

## Methods

### Derivation of iMSCs

The derivation of iMSCs was described in our previous studies [12, 13]. Briefly, one iPSC cell line, iPSCs-(C1P33), which was provided by the South China Institute for Stem Cell Biology and Regenerative Medicine Group of the Chinese Academy of Sciences in agreement with Professor Pei [23], was used to generate MSCs. After 5 days in culture, the medium was replaced by Dulbecco’s Modified Eagle Medium (DMEM) containing 10% fetal bovine serum (FBS), 2 mM l-glutamine, 1% penicillin/streptomycin (P/S), and 0.1 mM nonessential amino acids (all supplements from Gibco, Grand Island, NY, USA). Cells were passaged upon reaching approximately 80% confluence. After cells developed a homogeneous fibroblastic morphology, they were frozen at –80 °C for downstream experiments.

### Derivation of SMMSCs

The Ethics Committee of Shanghai Jiao Tong University Affiliated Sixth People’s Hospital approved the use of SMMSCs (Approval Number: YS-2016-063). Written informed consent was obtained from all donors. The SMMSC preparation method was described previously [24, 25]. In brief, synovium was harvested from three donors (two males/one female, age range 22–28 years) during anterior cruciate ligament (ACL) reconstruction surgery for acute ACL injuries. The harvested synovial membrane specimens were kept in high-glucose DMEM at 4 °C. Within 1 h, the specimen was rinsed with phosphate-buffered saline (PBS), finely minced, and digested with 0.2% collagenase I (Sigma–Aldrich, Saint Louis, MO, USA) in high-glucose DMEM containing 10% FBS and 1% P/S. After overnight incubation at 37 °C, the released cells were centrifuged, washed, resuspended in expansion medium (high-glucose DMEM supplemented with 10% FBS and 1% P/S), and plated in a T25 culture flask. The medium was changed after 4 days, and nonadherent cells were removed by thorough washing with PBS.

### Characterization of iMSCs and SMMSCs

Surface antigens of iMSCs and SMMSCs were analyzed by flow cytometry. Cells were harvested and incubated for 30 min with 3% bovine serum albumin (Gibco) in PBS to block nonspecific antigen binding. The iMSCs were then incubated with monoclonal antibodies against CD29, CD34, CD44, CD45, CD73 CD90, or HLA-DR; SMMSCs were incubated with monoclonal antibodies against CD34, CD44, CD45, CD73, CD90, CD166, or HLA-DR (all antibodies from BD Biosciences, Sparks Glencoe, MD, USA). The cells were then washed to remove unbound antibody. Surface antigens were analyzed using the Guava easyCyte™ flow cytometer (Millipore, Billerica, MA, USA).

### Isolation and identification of iMSC-Exos and SMMSC-Exos

iMSC-Exos and SMMSC-Exos were isolated and purified following our established protocol [13, 26]. After reaching 80% confluency, MSCs were washed with PBS and the culture medium was replaced with MesenGro hMSC medium (StemRD, San Francisco, CA, USA). The cells were then cultured for an additional 48 h at 37 °C in 5% CO_2_. The conditioned medium was collected and centrifuged at 300 × *g* for 10 min and then at 1500 × *g* for 10 min at 4 °C. After centrifugation, the supernatant was filtered using a 0.22-μm filter (Steritop™; Millipore) to remove the remaining cells and cellular debris. The supernatant was then transferred to an Ultra-clear tube (Millipore) and centrifuged at 4000 × *g* until the volume in the upper compartment was reduced to approximately 200 μl. The ultrafiltration liquid was resuspended in PBS and re-ultrafiltrated at 4000 × *g* to 200 μl. This step was then repeated once. Exosomes were stored in aliquots at –80 °C or used for other downstream experiments.

The concentration and size distribution of iMSC-Exos and SMMSC-Exos were measured using tunable resistive pulse sensing (TRPS) analysis by qNano (Izon Science, Cambridge, MA, USA). Aliquots of iMSC-Exos, SMMSC-Exos, or calibration particles (CPC100 particles; Izon Science) were placed in the Nanopore (NP150, A37355; Izon Science) at 47.0-mm stretch with a voltage of 0.6 V. Izon Control Suite software v2.2 (Izon Science) was used for data analysis. Exosome morphologies were observed using an FEI Tecnai G2 spirit transmission electron microscope (TEM; FEI, Eindhoven, the Netherlands). Antibodies against CD9 (1:1000; Abcam, Cambridge, UK), CD63 (1:1000; Abcam), and TSG101 (1:1000; Santa Cruz, Dallas, TX, USA) proteins were used to analyze the incorporation of each protein into exosomes in western blots.

### Collagenase-induced OA model

All procedures were approved by the Animal Research Committee of Shanghai Jiao Tong University Affiliated Sixth People’s Hospital (Approval Number: SYXK2011-0128). Six-week-old female C57B/L10 mice were randomized into four groups: normal (*n* = 5), iMSC-Exos treatment (*n* = 10), SMMSC-Exos treatment (*n* = 10), and OA (*n* = 10). On day 0, collagenase was used to induce OA in all mice in the iMSC-Exos, SMMSC-Exos, and OA treatment groups. The collagenase-induced model of OA was described previously [27, 28]. Mice were anesthetized by intraperitoneal injection of 10 ml/kg 4% chloral hydrate. The knee joints of the mice were injected once intra-articularly through the patellar ligament with 12 U of collagenase VII (*Clostridium histolyticum*; Sigma–Aldrich) in 8 μl saline. In the normal group, 8 μl of saline without collagenase was injected into the knee joints in the same way. On days 7, 14, and 21, mice in the iMSC-Exos and SMMSC-Exos treatment groups were injected intra-articularly with 8 μl iMSC-Exos (1.0 × 10^10^/ml) or 8 μl SMMSC-Exos in PBS (1.0 × 10^10^/ml), respectively. Mice in the OA and normal groups were injected intra-articularly with 8 μl PBS at each time point. On day 28, mice were euthanatized for further analysis.

### Macroscopic examination

After euthanasia, the surface of the proximal tibia was exposed. The surrounding soft tissue including joint capsule and meniscus was removed. The cartilage surface was then fully exposed and examined macroscopically. The evaluation was performed by two blinded investigators, and the score was based on the International Cartilage Research Society (ICRS) for cartilage repair [29].

### Histology

Mice tibias were fixed in 10% paraformaldehyde for 24 h and were then decalcified in 10% EDTA for 7 days at 37 °C. After serial dehydration, the tibial bones were embedded in paraffin and sectioned coronally through the tibial plateau at 5 μm thickness, and then stained with hematoxylin and eosin (H&E) and safranin O/fast green. Each specimen was scored for the medial tibial plateau by two blinded observers using the Osteoarthritis Research Society International (OARSI) cartilage OA histopathology grading system to histologically grade the severity of cartilage destruction [30].

### Immunohistochemistry analysis

Immunohistochemical (IHC) staining for type I and II collagens was performed. All sections were deparaffinized, washed with PBS, treated for antigen retrieval, and blocked with mouse IgG for 30 min. Sections were incubated with primary antibodies against mouse anti-collagen I (1:200; Abcam) and mouse anti-collagen II (1:200; Abcam) overnight at 4 °C. Biotinylated secondary antibody and streptavidin peroxidase solution were then used to visualize the sections.

### Chondrocyte migration assay

Human cartilage was harvested after obtaining informed consent from donors. Chondrocyte preparation was described previously [25]. The scratch wound assay was used to analyze the effect of iMSC-Exos and SMMSC-Exos on migration of chondrocytes, as described previously [13]. Briefly, 1.5 × 10^4^ cells were seeded into 12-well plates and maintained at 37 °C for 8 h. Next, the confluent monolayer of cells was scratched using the tip of a P200 pipet tip. The medium was removed and the cells were washed once with PBS. The medium was then replaced with fresh DMEM F-12 medium containing 10^8^/ml iMSC-Exos, 10^8^/ml SMMSC-Exos, or control medium. Wound closure was monitored by collecting digital images at 0, 24, and 48 h after the scratch using an inverted microscope (Leica, Wetzlar, Germany). The images were obtained at the same position before and after incubation. Scratched areas were measured using Image-Pro Plus 6.0 software (Media Cybernetics, Bethesda, MD, USA).

### Chondrocyte proliferation assay

The effect of iMSC-Exos and SMMSC-Exos on the proliferation of human chondrocytes was evaluated using the Cell Counting Kit-8 (CCK-8; Dojindo, Kyushu Island, Japan) as described previously [12, 13]. Chondrocytes were seeded into 96-well plates at 2 × 10^3^ cells/well. After 8 h, different doses of iMSC-Exos or SMMSC-Exos were added to the wells. The medium was changed daily for 5 days, using fresh DMEM F-12 medium containing 10% FBS and the same exosome concentrations. Cell proliferation curves were constructed by measuring the amount of formazan dye generated by cellular dehydrogenase activity with a microplate reader at a wavelength of 450 nm.

### Statistical analysis

The data were presented as means ± standard deviation. Comparisons of macroscopic and histological scores as well as scratch wound assay results were made using the Mann–Whitney *U* test. Comparisons of chondrocyte proliferation assays were performed using unpaired Student’s *t* test. *P* < 0.05 was considered statistically significant.

## Results

### Characterization of iMSCs and SMMSCs

iMSCs were successfully derived from iPSCs using our modified one-step induction protocol. More than 90% of iMSCs showed a homogeneous fibroblastic morphology after cells were passaged through four or five propagations. After primary culture and throughout in-vitro expansion, SMMSCs showed a robust proliferation capability and appeared to be a relatively homogeneous population of spindle-shaped cells. The trilineage differentiation capacity of SMMSCs was presented in Additional file 1: Figure S1.

**Fig. 1:**
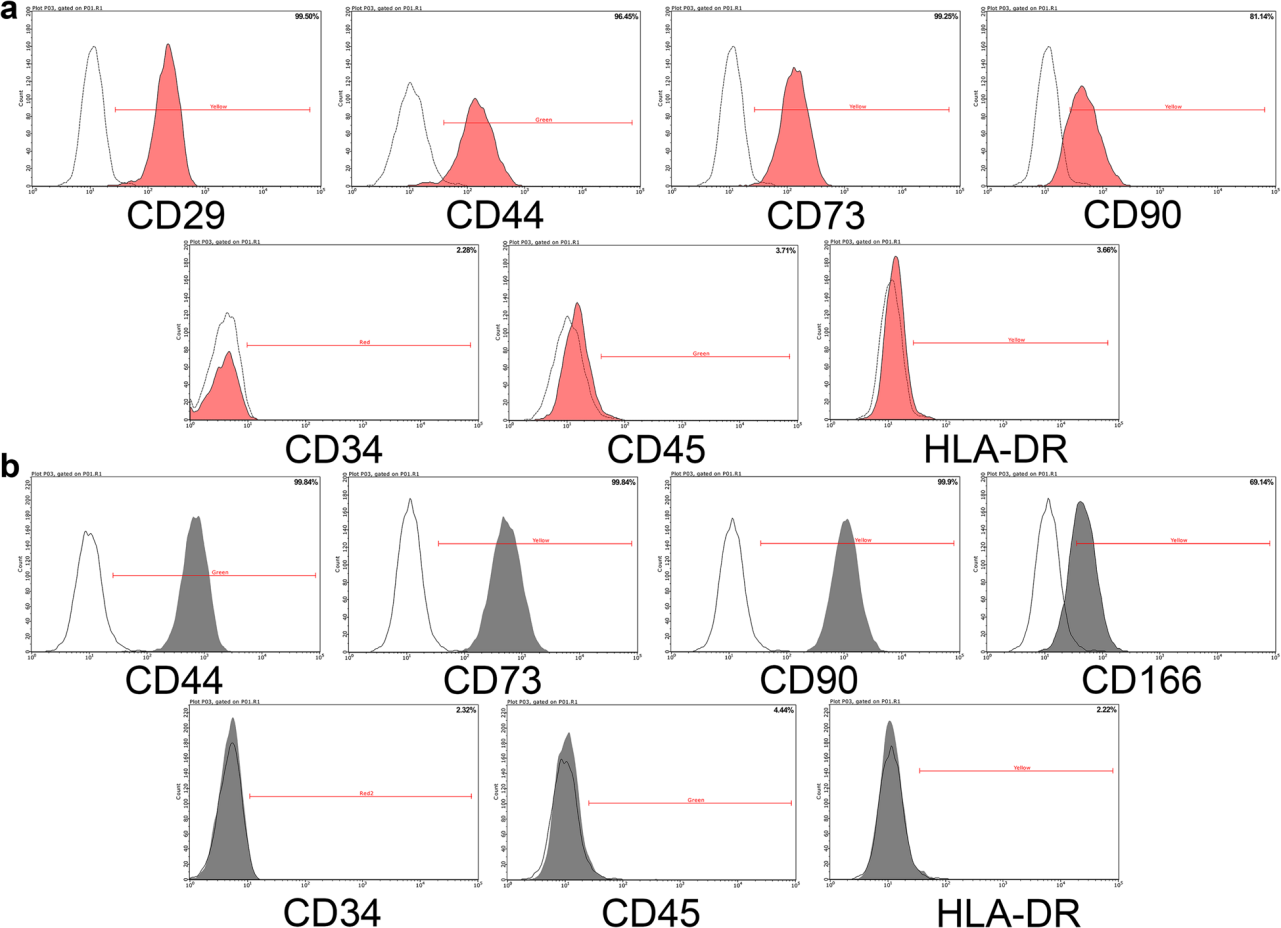
Flow cytometric analyses of phenotypic markers of iMSCs and SMMSCs. **a** iMSCs were positive for CD29, CD44, CD73, and CD90 and were negative for CD34, CD45, and HLA-DR. **b** SMMSCs were positive for CD44, CD73, CD90, and CD166 and were negative for CD34, CD45, and HLA-DR

Flow cytometric analysis demonstrated that the majority of iMSCs expressed CD29, CD44, CD73, and CD90 and were negative for CD34, CD45, and HLA-DR (Fig. 1a). The majority of SMMSCs expressed CD44, CD73, CD90, and CD166 and were negative for CD34, CD45, and HLA-DR (Fig. 1b).

### Characterization of iMSC-Exos and SMMSC-Exos

**Fig. 2:**
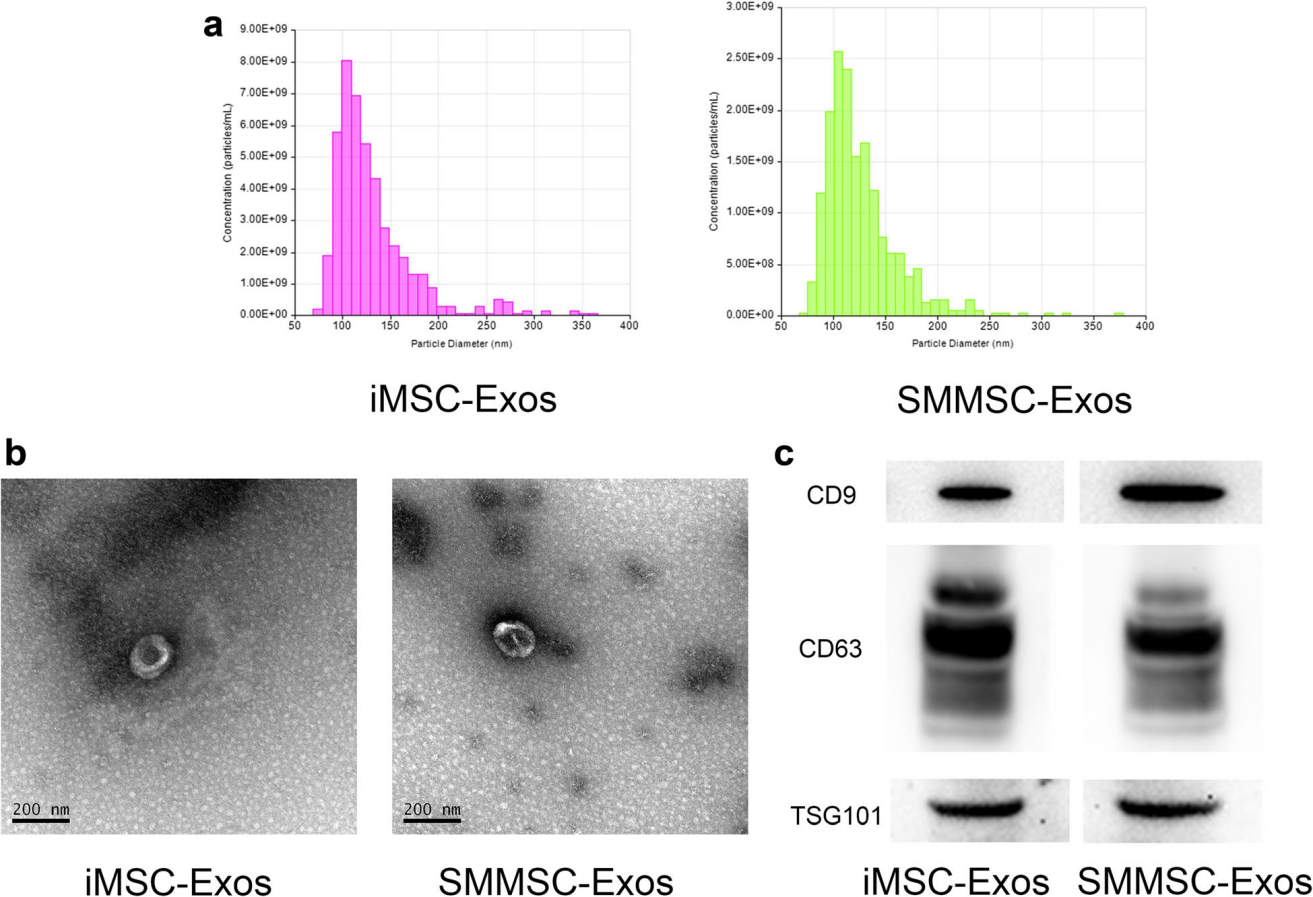
Characterization of iMSC-Exos and SMMSC-Exos. **a** TRPS measurement of exosome concentration and size distribution. **b** Morphology of exosomes under transmission electron microscopy. **c** Western blot analysis of exosome-specific CD9, CD63, and TSG101 proteins. *iMSC-Exos* exosomes secreted by induced pluripotent stem cell-derived mesenchymal stem cells, *SMMSC-Exos* exosomes secreted by synovial membrane mesenchymal stem cells

qNano analysis showed that the size of the majority of iMSC-Exos and SMMSC-Exos was approximately 50–150 nm (Fig. 2a). Transmission electron microscopy clearly revealed that iMSC-Exos and SMMSC-Exos exhibited a cup-shaped or round-shaped morphology with a diameter of 50–200 nm (Fig. 2b). Western blotting analyses indicated that the iMSC-Exos and SMMSC-Exos expressed exosomal markers such as CD9, CD63, and TSG101 proteins (Fig. 2c).

### Macroscopic examination

**Fig. 3:**
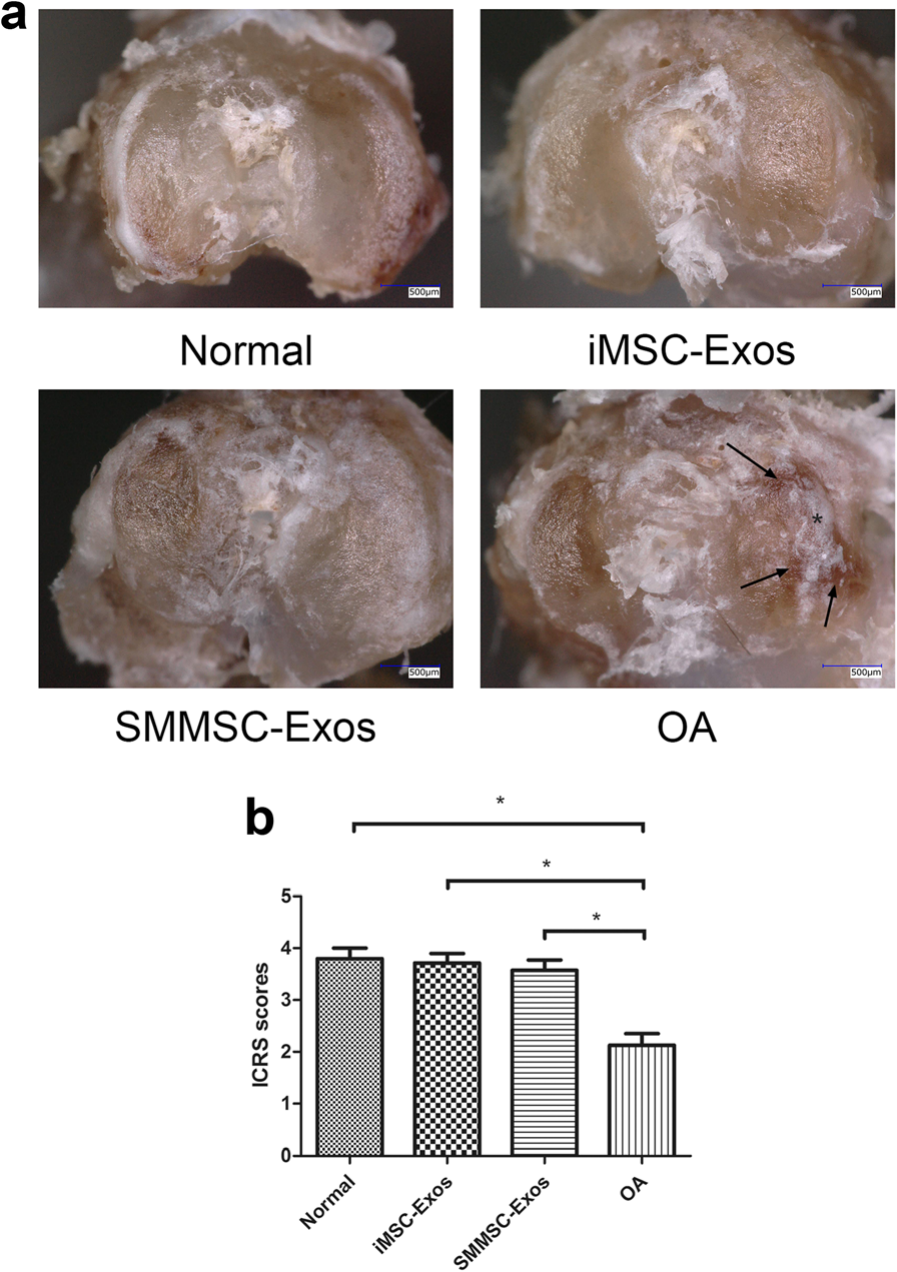
Macroscopic examination of tibial plateaus. **a** Representative macroscopic images of the tibial plateau. Changes representative of OA were observed only in the OA group. *Black arrows*, subchondral bone exposure; *asterisks*, surface fibrillation. **b** Macroscopic ICRS scores showed that the normal, iMSC-Exos, and SMMSC-Exos groups had significantly higher scores compared with the OA group. *P < 0.05. *ICRS* International Cartilage Research Society, *iMSC-Exos* exosomes secreted by induced pluripotent stem cell-derived mesenchymal stem cells, *OA* osteoarthritis, *SMMSC-Exos* exosomes secreted by synovial membrane mesenchymal stem cells

The gross appearance of the tibial plateau was evaluated in each group. The joint surface of the OA group showed marked gross changes in OA, including cartilage abrasion, subchondral bone exposure, and surface fibrillation (Fig. 3a). Analysis of the ICRS scores revealed no significant differences among the normal, iMSC-Exos, and SMMSC-Exos groups. However, these three groups had significantly higher ICRS scores compared with the OA group (Fig. 3b).

### Histological analysis

**Fig. 4:**
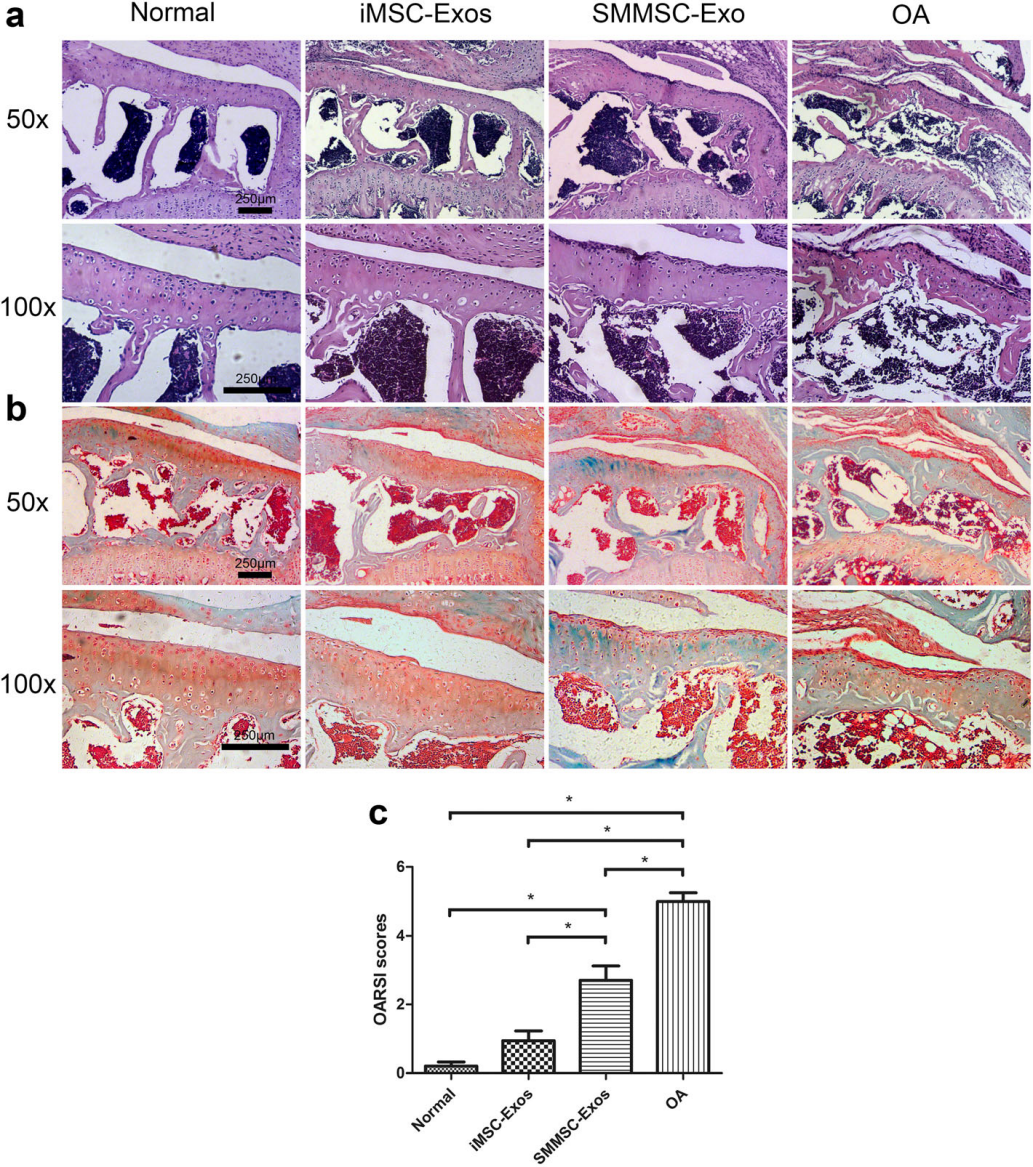
Histological analysis. **a** H&E staining. **b** Safranin O/fast green staining. H&E and safranin O/fast green staining showed that the normal group and iMSC-Exos group presented typical hyaline features with a smooth cartilage surface, regular cellular organization, and normal proteoglycan content. Compared with the iMSC-Exos group, the SMMSC-Exos group showed moderate surface irregularity, superficial fibrillation, and a loss of proteoglycan (*reddish-orange* stain). **c** OARSI scores in the normal, iMSC-Exos, and SMMSC-Exos groups were significantly lower than in the OA group. The score of the iMSC-Exos group was significantly lower than the SMMSC-Exos group, while there was no significant difference between the iMSC-Exos group and the normal group. *P < 0.05. *iMSC-Exos* exosomes secreted by induced pluripotent stem cell-derived mesenchymal stem cells, *OA* osteoarthritis, *OARSI* Osteoarthritis Research Society International, *SMMSC-Exos* exosomes secreted by synovial membrane mesenchymal stem cells

Cartilage tissues from the medial tibial plateau in the normal group and the iMSC-Exos group presented typical hyaline features with a smooth cartilage surface, regular cellular organization, and normal proteoglycan content (Fig. 4a, left panels). However, the OA group showed typical degenerative OA changes including fibrillation of the articular surface, proteoglycan depletion, osteophytic remodeling, and articular cartilage reduction (Fig. 4a, right panel). Compared with the iMSC-Exos group, animals treated with SMMSC-Exos showed moderate surface irregularity and superficial fibrillation. In safranin O/fast green sections (Fig. 4b), a reduction in safranin O staining was also noted in the SMMSC-Exos group compared with the iMSC-Exos group, which indicated a loss of proteoglycan in cartilage in the SMMSC-Exos group. The OARSI scores in the normal, iMSC-Exos, and SMMSC-Exos groups were significantly lower than in the OA group (Fig. 4c). The score of the iMSC-Exos group was significantly lower than that of SMMSC-Exos group, but there was no significant difference between the iMSC-Exos and normal groups.

### IHC analysis

**Fig. 5:**
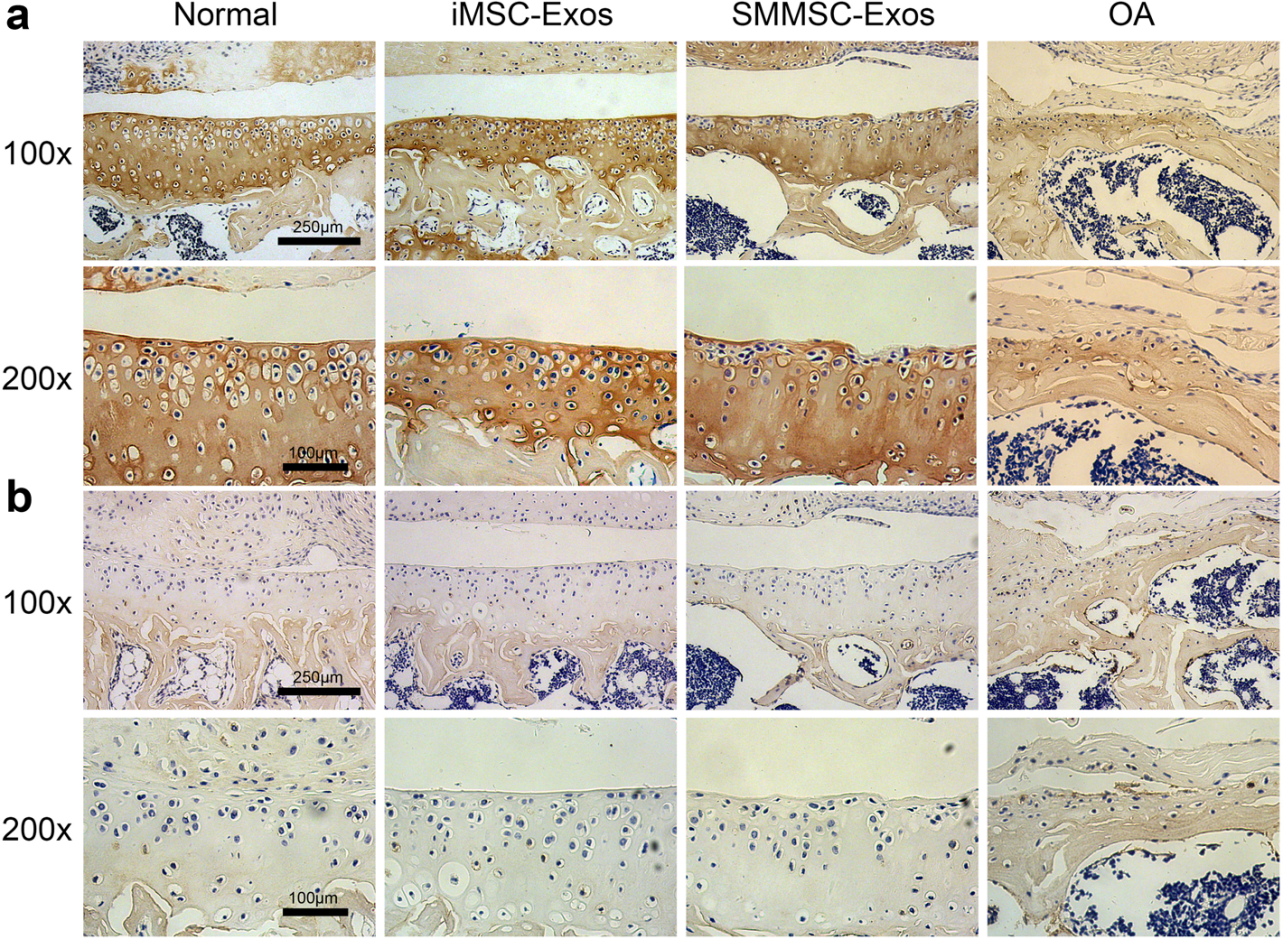
IHC analysis. **a** Collagen II staining. Staining of cartilage (*brown* coloration) in the normal, iMSC-Exos, and SMMSC-Exos groups was stronger than in the OA group. In the SMMSC-Exos group, collagen II expressed very weakly at the superficial zone compared with the iMSC-Exos group. **b** Collagen I staining. Collagen I expression was not found in the cartilage of the normal, iMSC-Exos, and SMMSC-Exos groups, but was present in the OA group. *iMSC-Exos* exosomes secreted by induced pluripotent stem cell-derived mesenchymal stem cells, *OA* osteoarthritis, *SMMSC-Exos* exosomes secreted by synovial membrane mesenchymal stem cells

IHC analysis of articular cartilage revealed that collagen II staining in the normal, iMSC-Exos, and SMMSC-Exos groups was more intense than in the OA group (Fig. 5a). In the normal and iMSC-Exos groups, collagen II staining was localized primarily to the superficial and deep zones of cartilage. In the SMMSC-Exos group, collagen II expressed very weakly at the superficial zone compared with the iMSC-Exos group. Collagen I staining of cartilage was not observed in the normal, iMSC-Exos, and SMMSC-Exos groups, but was present in the OA group (Fig. 5b).

### Chondrocyte migration and proliferation assays

**Fig. 6:**
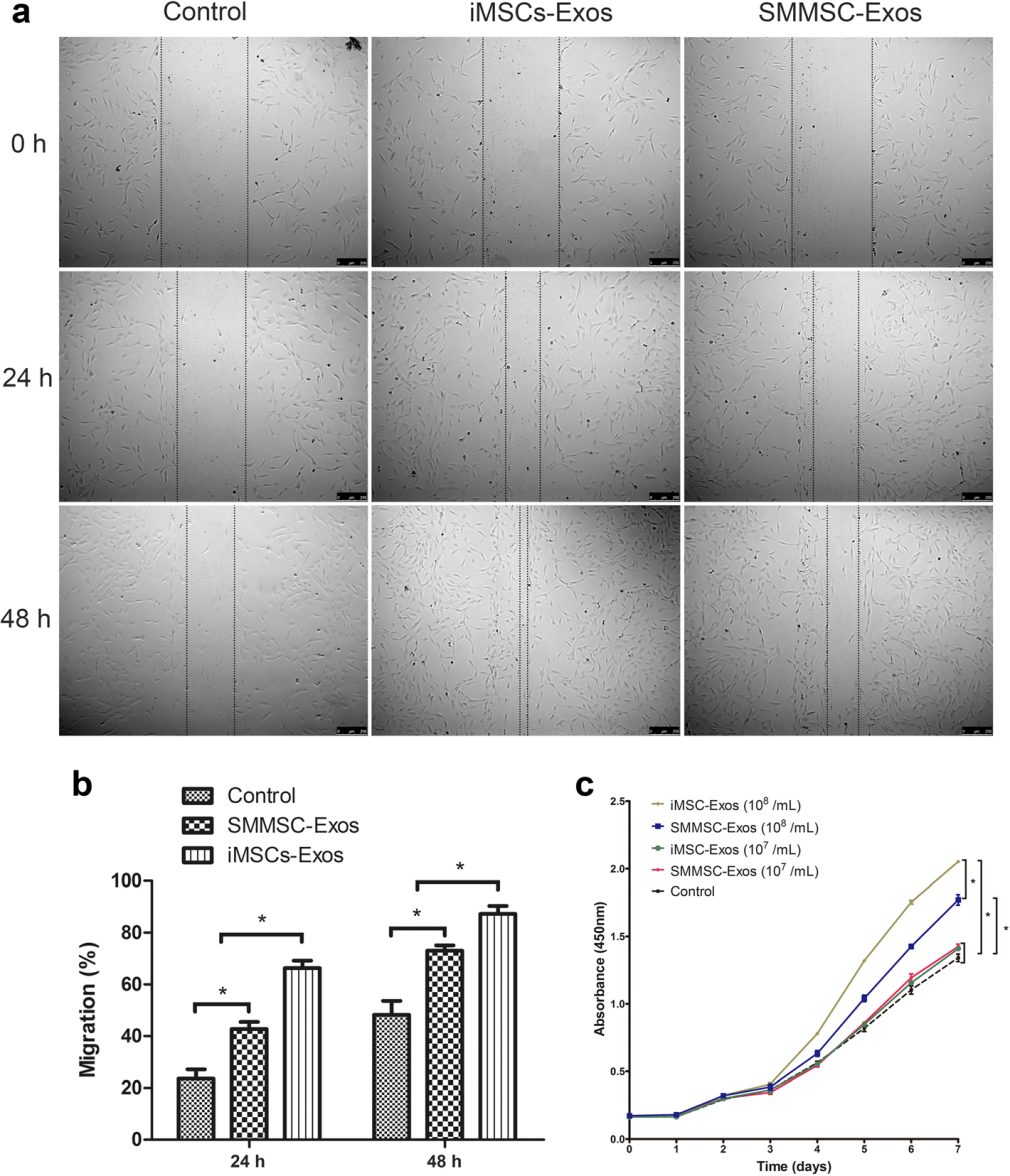
Effects of iMSC-Exos and SMMSC-Exos on migration and proliferation of chondrocytes. **a** Light microscopy images of scratch wound assays. **b** Quantitative analysis of migration rates at 24 and 48 h. Scratch wound assays indicated that both iMSC-Exos and SMMSC-Exos significantly enhanced the motility of chondrocytes and that iMSC-Exos were more effective than SMMSC-Exos. **c** iMSC-Exos and SMMSC-Exos stimulated chondrocyte proliferation in a dose-dependent manner. At the concentration of 10^8^ exosomes/ml, iMSC-Exos showed a more powerful effect on chondrocyte proliferation than did SMMSC-Exos. *P < 0.05. *iMSC-Exos* exosomes secreted by induced pluripotent stem cell-derived mesenchymal stem cells, *OARSI* Osteoarthritis Research Society International, *SMMSC-Exos* exosomes secreted by synovial membrane mesenchymal stem cells

Scratch wound assays indicated that both iMSC-Exos and SMMSC-Exos significantly enhanced the motility of chondrocytes (*P* < 0.05) and further showed that iMSC-Exos were more effective than SMMSC-Exos in increasing motility at 24 and 48 h (*P* < 0.05; Fig. 6a, b).

iMSC-Exos and SMMSC-Exos stimulated chondrocyte proliferation in a dose-dependent manner. At the concentration of 10^8^ exosomes/ml, chondrocytes cultured with either iMSC-Exos or SMMSC-Exos showed greater proliferation compared with the control group or with groups treated with 10^7^ exosomes/ml, and iMSC-Exos had a more potent effect on chondrocyte proliferation than SMMSC-Exos. However, at a concentration of 10^7^ exosomes/ml, there were no significant differences among the iMSC-Exos, SMMSC-Exos, and control groups (Fig. 6c).

## Discussion

In the present study, we compared for the first time the effect of exosomes derived from iMSCs and SMMSCs on the treatment of OA. The injection of either iMSC-Exos or SMMSC-Exos attenuated OA in a mouse collagenase-induced OA model, but iMSC-Exos had a superior therapeutic effect compared with SMMSC-Exos. Furthermore, we demonstrated that while iMSC-Exos and SMMSC-Exos both stimulated chondrocyte migration and proliferation, iMSC-Exos had a greater effect than SMMSC-Exos.

iPSCs and ESCs are pluripotent stem cells. iPSC-derived and ESC-derived MSCs have been reported as promising therapies for treating various tissue injuries like bone defects, hepatic failure, and myocardial and limb ischemia [22, 31, 32]. Recently, Gibson et al. [33] demonstrated that BMP-2 and Wnt5a-pretreated ESC-derived MSCs could promote rat chondral defect repair. Similar to direct MSC transplantation therapy, our previous studies indicated that iMSC-Exos also have the therapeutic effect of facilitating cutaneous wound healing [12], attenuating limb ischemia [13], and enhancing bone regeneration [34]. However, the effect of iMSC-Exos on OA repair has not been reported in the literature. In the present study, we found that injection of iMSC-Exos significantly attenuated OA in a mouse model of collagenase-induced OA. Histological analysis demonstrated that the repaired cartilage in the iMSC-Exos group presented typical hyaline features similar to normal cartilage. IHC analysis indicated that expression of collagen II, a specific marker of hyaline cartilage, was similar in the iMSC-Exos and normal control groups. Further in-vitro study showed that iMSC-Exos produced significant increases in chondrocyte migration and proliferation.

SMMSCs are derived from the synovial membrane and possess high self-renewal capacity [24]. Previous studies demonstrated that the synovium and articular cartilage develop from a common population of cells during the development of synovial joints [17], so SMMSCs are developmentally more closely related to chondrocytes than other MSCs. SMMSCs were also reported to have a greater capacity to stimulate chondrogenesis than BMSCs and AMSCs, making them more suitable for cartilage repair [19]. Recent studies have highlighted the role of SMMSCs for the treatment of OA, and one study reported that SMMSCs inhibited OA progression in rats [16]. However, the effect of SMMSC-Exos on OA repair has not been reported. The present study demonstrated that the injection of SMMSC-Exoscould significantly attenuate OA progression in a mouse collagenase-induced OA model. However, IHC analysis showed only weak collagen II expression at the superficial zone in the SMMSC-Exos group compared with normal cartilage. Further in-vitro study indicated that, similar to iMSC-Exos, SMMSC-Exos could also significantly stimulate chondrocyte migration and proliferation.

Using the same number of exosomes, iMSC-Exos exerted a superior therapeutic effect compared with SMMSC-Exos in the mouse OA model. Cartilage treated by SMMSC-Exos showed moderate surface irregularity, superficial fibrillation, loss of proteoglycan, and loss of cartilage in the superficial zone, but none of these conditions were observed when cartilage was treated with iMSC-Exos. Furthermore, iMSC-Exos had a stronger effect than SMMSC-Exos on chondrocyte migration and proliferation. In addition to these therapeutic advantages, there are several features that make iMSCs worth considering as a source of exosomes. First, the harvesting of iMSCs can be performed noninvasively. iPSCs can be induced from patient-specific adult somatic cells such as peripheral blood cells, in contrast to the harvesting procedure for SMMSCs from synovial membrane which requires an invasive surgical procedure. Second, transplantation of patient-specific iMSCs can theoretically overcome potential problems related to ethical issues and the need for immunosuppression in the recipient. Third, autologous iMSCs may provide an inexhaustible source of MSCs that could be used to meet unmet clinical needs. Most importantly, iMSCs are emerging as a strong contender for a new source of MSCs that would be suitable to replace adult MSCs.

Exosomes contain many regulatory signals such as RNAs, microRNAs, and proteins, which may be a key mechanism underlying their ability to alter cellular signaling, reduce inflammation, and induce tissue repair [35, 36]. Li et al. [37] reported that human umbilical cord MSC-derived exosomes could attenuate burn-induced inflammation mediated by miR-181c. Xin et al. [38] demonstrated that BMSC-derived exosomes could promote neural plasticity and functional recovery in a rat stroke model via transfer of miR-133b. Zhang et al. [39] showed that exosomal 14-3-3ζ protein from human umbilical cord MSCs plays an important role in cutaneous regeneration. Although the precise mechanism of exosomes in OA repair is still unclear, we speculate that one or more components such as microRNAs or proteins may play a crucial role. Future work will need to focus on the components present in MSC exosomes which participate in OA repair and their mechanism of action.

## Conclusions

The present study demonstrated that iMSC-Exos had a greater therapeutic effect than SMMSC-Exos in an experimental mouse model of collagenase-induced OA. Similarly, iMSC-Exos exerted a stronger stimulatory effect on chondrocyte migration and proliferation than did SMMSC-Exos. Because iMSCs can be obtained in a patient-specific manner and are theoretically inexhaustible, iMSC-Exos may represent a novel therapeutic approach for the treatment of OA.

## Additional file

Additional file 1: Is **Figure S1.** showing the trilineage differentiation capacity of SMMSCs. (**A**) Alizarin Red staining for osteogenic mineralization after 4 weeks in culture with osteogenic medium. (**B**) Oil Red O staining for small lipid droplets after 3 weeks in culture with adipogenic medium. (**C**) Alcian Blue staining for cartilaginous extracellular matrix after 4 weeks in culture with chondrogenic medium. (TIF 9506 kb)

## Abbreviations

ACL: Anterior cruciate ligament
AMSC: Adipose-derived mesenchymal stem cell
BMSC: Bone marrow-derived MSC
DMEM: Dulbecco’s Modified Eagle Medium
ESC: Embryonic stem cell
H&E: Hematoxylin and eosin
ICRS: International Cartilage Research Society
iMSC: Induced pluripotent stem cell-derived mesenchymal stem cell
iMSC-Exos: Exosomes secreted by induced pluripotent stem cell-derived mesenchymal stem cells
iPSC: Induced pluripotent stem cell
MSC: Mesenchymal stem cell
OA: Osteoarthritis
OARSI: Osteoarthritis Research Society International
SMMSC: Synovial membrane-derived MSC
SMMSC-Exos: Exosomes secreted by synovial membrane mesenchymal stem cells
TRPS: tunable resistive pulse sensing
