# Supplementary figures and images for: Comparison of exosomes secreted by induced pluripotent stem cell-derived mesenchymal stem cells and synovial membrane-derived mesenchymal stem cells for the treatment of osteoarthritis
Source: Stem Cell Res Ther. 2017 Mar 9;8:64. doi: 10.1186/s13287-017-0510-9 (PMC5345222; doi:10.1186/s13287-017-0510-9)

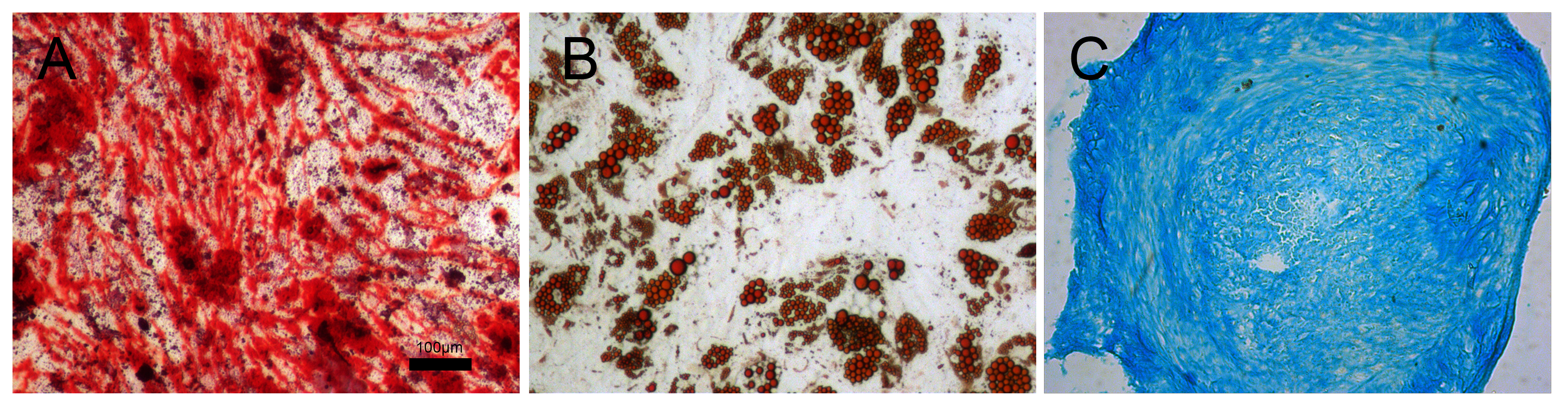

Supplement: Additional file 1: — Is Figure S1. showing the trilineage differentiation capacity of SMMSCs. (A) Alizarin Red staining for osteogenic mineralization after 4 weeks in culture with osteogenic medium. (B) Oil Red O staining for small lipid droplets after 3 weeks in culture with adipogenic medium. (C) Alcian Blue staining for cartilaginous extracellular matrix after 4 weeks in culture with chondrogenic medium. (TIF 9506 kb) [file 13287_2017_510_MOESM1_ESM.tif]
